# Implications of draxin in neurological disorders

**DOI:** 10.3389/fcell.2025.1560940

**Published:** 2025-02-24

**Authors:** Yohei Shinmyo

**Affiliations:** Department of Neurophysiology, Hamamatsu University School of Medicine, Shizuoka, Japan

**Keywords:** axon guidance, draxin, BTBR mouse, ASD, corpus callosum

## Abstract

Axon guidance proteins not only play a role in the formation of proper neural circuits but also have other important functions, such as cell survival, migration, and proliferation in the brain. Therefore, mutations in the genes encoding these proteins frequently cause various types of neurological disorders, including psychiatric disorders and neurodegenerative diseases. We previously identified an axon guidance protein, draxin, that is essential for the development of several neural circuits and cell survival in the brain. Recently, the deletion of the *draxin* gene was identified in an inbred BTBR T^+^ Itpr3^tf^/J (BTBR/J) mouse, which is a widely used model of Autism Spectrum Disorder (ASD), suggesting that *draxin* deletion is a genetic factor for ASD-like characteristics in BTBR/J mice. In this review, I summarize the neuroanatomical abnormalities in *draxin* knockout mice by comparing them to BTBR/J mice and discuss the possible contributions of draxin to anatomical and behavioral phenotypes in BTBR/J mice.

## Introduction

Draxin was first identified as an axon guidance protein that regulates commissural axons in the spinal cord and the forebrain. It is a secreted protein that shares no homology with other known proteins (Islam et al., 2009; Miyake et al., 2009). Draxin has been shown to bind to netrin-1 and its receptors, including Deleted in colorectal cancer (Dcc) and Neogenin (Neo1) (Ahmed et al., 2011; Shinmyo et al., 2015). Previous studies have suggested that draxin regulates the outgrowth of axons originating from various types of neurons *in vitro* (Islam et al., 2009; Naser et al., 2009; Ahmed et al., 2010; Ahmed et al., 2011; Chen et al., 2013; Meli et al., 2015; Shinmyo et al., 2015). *Draxin* knockout (KO) mice show developmental abnormalities in various neural circuits, including the corpus callosum, the hippocampal commissure, the anterior commissure, the fornix, and the thalamocortical axons (Islam et al., 2009; Zhang et al., 2010; Shinmyo et al., 2015). Thus, draxin may control the development of neural circuits in the brain through the netrin-1 receptors or by modulating netrin-1-mediated axon guidance.

Previous human and animal studies have shown that axon guidance proteins are associated with structural changes in neuronal connections during neurological disorders (Nugent et al., 2012; Van Battum et al., 2015). In addition, because axon guidance cues have other important functions in the brain, such as cell survival, migration, and proliferation (Mehlen et al., 2011), mutations in the genes encoding axon guidance proteins can cause many neurological disorders. Indeed, draxin and/or netrin signaling has been shown to be associated with several neurological disorders, including psychiatric disorders, gliomas, and neurodegenerative diseases (Infante et al., 2015; Vosberg et al., 2020; Ahn et al., 2021; Jasmin et al., 2021; Cai et al., 2024). Recently, an 8-bp frameshift deletion of the *draxin* gene was identified in an inbred BTBR T^+^ Itpr3^tf^/J (BTBR/J) mouse, a widely used model of Autism Spectrum Disorder (ASD) (Morcom et al., 2021; Arslan et al., 2023). Furthermore, *draxin* deletion in BTBR/J mice was shown to contribute to the dysgenesis of the corpus callosum, which is a neuroanatomical abnormality characteristic of human ASD (Arslan et al., 2023). In this review, I summarized the neuroanatomical abnormalities in *draxin* KO mice by comparing them to BRBR/J mice.

### Dysgenesis of the corpus callosum in human ASD

ASD is a neurodevelopmental disorder defined by impairments in social interactions, communication deficits, and repetitive behaviors with restricted interests (Lai et al., 2014). Identifying abnormalities in brain structures in ASD is critical for developing more precise and objective diagnoses and for creating effective new treatments. One prominent mechanism that has been suggested to contribute to the underlying pathology of ASD is abnormal long-range neuronal connectivity. This is because numerous MRI studies have demonstrated reduced fractional anisotropy in major white matter tracts in individuals with ASD, including the cingulum, uncinate fasciculi, occipitotemporal tracts, and, most consistently, the corpus callosum (Barnea-Goraly et al., 2004; Alexander et al., 2007; Keller et al., 2007; Frazier and Hardan, 2009; Kumar et al., 2010; Weinstein et al., 2011).

The corpus callosum is a large bundle of nerve fibers that connects the left and right hemispheres of the brain. Variable corpus callosum abnormalities have been reported in the anterior, midbody, and posterior regions of the forebrain in ASD (Egaas et al., 1995; Saitoh et al., 1995; Haas et al., 1996; Piven et al., 1997; Manes et al., 1999; Hardan et al., 2000). These observations suggest that the abnormal development of the corpus callosum is associated with ASD. This is consistent with recent results from mega-analyses comparing white matter microstructural differences between healthy participants and those with psychiatric disorders, showing that patients with schizophrenia, bipolar disorder, or ASD disorder have common alterations in the corpus callosum (Koshiyama et al., 2020).

The corpus callosum plays a critical role in the transmission and integration of information between the left and the right hemispheres. The anterior corpus callosum connects regions of the prefrontal cortex and is associated with higher-order cognitive, emotional, and social functions. The midbody of the corpus callosum connects multiple regions, including the primary motor and sensory cortices, and is involved in sensory and motor processing. The posterior corpus callosum links the occipital lobes and is crucial for the processing and integration of visual information. Abnormal development in specific regions of the corpus callosum may be associated with the specific cognitive and behavioral characteristics of ASD. However, abnormalities in brain structures in patients with ASD have been observed not only in the corpus callosum but also in other regions. Therefore, to understand the causes of behavioral abnormalities in ASD accurately, it is important to analyze animal models of specific anatomical and functional abnormalities.

### BTBR mouse, an idiopathic animal model of ASD

Characteristic behavioral phenotypes of ASD have been modeled in mice. One such model is the inbred BTBR/J mouse, which is the most extensively researched and the most commonly reproduced inbred strain (Nadler et al., 2006; Bolivar et al., 2007; Moy et al., 2007). BTBR/J mice exhibit impaired in social interactions and high levels of repetitive behaviors (Moy et al., 2007; McFarlane et al., 2008; Dodero et al., 2013). Furthermore, this strain is characterized by the absence of the corpus callosum and a smaller-to-absent hippocampal commissure (Wahlsten et al., 2003). A previous study identified several genomic regions in BTBR/J mice that distinctly influenced their ASD-like characteristics (Jones-Davis et al., 2013). Recently, an 8-bp frameshift deletion of the *draxin* gene, leading to the loss of draxin function, was identified in BTBR/J mice (Morcom et al., 2021; Arslan et al., 2023). The *draxin* gene is located in a genomic region that was previously identified as contributing to commissural abnormalities in BTBR/J mice (Jones-Davis et al., 2013). Since *draxin* KO mice display malformations of the corpus callosum and the hippocampal commissure, draxin is a promising candidate for explaining the defects in these commissures in BTBR/J mice. Consistently, abnormal development of the corpus callosum was partially restored in BTBR/J mice with a heterozygous knock-in that reverted the 8 bp *draxin* deletion to the wild-type, suggesting that the draxin deletion contributes to agenesis of the corpus callosum in BTBR/J mice (Arslan et al., 2023).

### Similarities in neuroanatomical phenotypes between *draxin* KO and BTBR mice

Since previous studies have suggested that BTBR/J mice are characterized by multiple genetic aberrations, it is important to clarify the contribution of draxin to the anatomical and behavioral phenotypes of BTBR/J mice. *Draxin* KO mice show various developmental abnormalities in the brain similar to those observed in BTBR/J mice. BTBR/J mice exhibit an absence of the corpus callosum, and reductions in the hippocampal and the anterior commissures (Table 1) (Wahlsten et al., 2003; Ellegood et al., 2015). Similar to BTBR/J mice, *draxin* KO mice show severe defects in all forebrain commissures, the corpus callosum, the hippocampal commissure, and the anterior commissure (Islam et al., 2009). Given that the abnormal development of the corpus callosum was partially rescued in BTBR/J mice with a heterozygous knock-in that reverted the 8 bp *draxin* deletion to the wild-type, the *draxin* deletion contributes to the absence of the corpus callosum in BTBR/J mice (Arslan et al., 2023). However, this observation suggests that additional genetic factors contribute to the absence of the corpus callosum in BTBR/J mice. Both *draxin* KO mice and BTBR mice with a C57Bl/6J genetic background display variable penetrance of the corpus callosum defect, suggesting that other genetic factors modify the corpus callosum phenotype driven by the *draxin* mutation (Morcom et al., 2021).

**TABLE 1:** Anatomical abnormalities in brains of *draxin* KO and BTBR mice.

	*Draxin* KO	BTBR/J
Aberrant neural circuits		
Corpus callosum	+	+
Hippocampal commissure	+	+
Anterior commissure	+	+
Thalamocortical axons	+	+
Corticofugal axons	+	?
Fornix	+	?
Other abnormalities in the brain		
Shrinkage of the hippocampus	+	+
Reduced size of the amygdala	?	+

+ Abnormal development; ?, not investigated.

*Draxin* KO mice also show severe defects in the thalamocortical and corticofugal projections (Shinmyo et al., 2015). During normal brain development, corticofugal and thalamocortical axons meet in the internal capsule and depend on each other for their guidance to the thalamus and neocortex, respectively (Lopez-Bendito and Molnar, 2003). Corticofugal axons grow from the cortex into the internal capsule in wild-type mice. In contrast, some corticofugal axons of *draxin* KO mice do not enter the internal capsule but instead grow toward the external capsule. Thalamocortical axons in *draxin* KO mice grow normally toward the internal capsule. However, some of them do not enter the cortex and instead either stall or turn laterally toward the external capsule, whereas others enter the cortex with an abnormal topographic organization. Visualization of the cortical sensory regions revealed disruptions in the spatial positions of thalamocortical axon terminals in *draxin* KO mice (Shinmyo et al., 2015). Thus, draxin is essential for guiding thalamocortical axons from the internal capsule to the cortex, as well as for their region-specific connections between the thalamus and cortex. Importantly, the topography of thalamocortical projections changes in BTBR/J mice, in which the primary somatosensory and visual cortical areas are medially shifted (Fenlon et al., 2015). Therefore, abnormalities in the topographic organization of thalamocortical projections are a common feature of *draxin* KO and BTBR/J mice, although this phenotype in *draxin* KO mice requires further investigation. Another similarity in the anatomical phenotype between *draxin* KO mice (Zhang et al., 2010) and BTBR/J mice (Mercier et al., 2012) is the shrinkage of the hippocampus. In addition to the hippocampus, the size of the amygdala nuclei is reduced in BTBR/J mice (Mercier et al., 2012). However, it remains unclear whether the anatomy of the amygdala is altered in *draxin* KO mice or not. Collectively, *draxin* deletion is likely to be the primary genetic factor underlying the neuroanatomical phenotypes in BTBR/J mice.

## Discussion

In this review, I have summarized the similarities in neuroanatomical phenotypes between *draxin* KO and BTBR/J mice. In addition to their phenotypical similarities, recent studies have suggested that draxin contributes to neuroanatomical phenotypes in BTBR/J mice (Morcom et al., 2021; Arslan et al., 2023). However, the contribution of draxin to the behavioral phenotypes of BTBR/J mice remains unclear. To address this issue, it is necessary to perform behavioral analyses in *draxin* KO mice and *draxin* knock-in BTBR mice.

It is important to determine the neuroanatomical abnormalities responsible for the behavioral phenotypes of ASD. Previous studies on humans with ASD and BTBR/J mice have suggested that dysgenesis of the corpus callosum is strongly associated with behavioral abnormalities in ASD. However, there is no direct evidence supporting this idea because dysgenesis of the corpus callosum is generally accompanied by other anomalies in brain structures in both humans and mice. For example, patients with corpus callosum anomalies frequently display dysgenesis of the hippocampal commissure (Hetts et al., 2006). Therefore, to examine whether the behavioral phenotypes characteristic of ASD are caused by anomalies in the corpus callosum, a mouse model with a specific defect in the corpus callosum is required. Surgical lesions of the corpus callosum at an early postnatal stage do not affect the juvenile play or adult social behaviors, nor do they increase repetitive self-grooming (Yang et al., 2009). This evidence does not support the hypothesis that disconnection of the corpus callosum is a causal factor for ASD-like behaviors in mice. However, experimental lesions at the postnatal stage may not replicate congenital corpus callosum anomalies. Both BTBR/J and *draxin* KO mice show corpus callosum agenesis with similar misprojections of the callosal axons. In these mice, callosal axons fail to cross the midline; instead, they form ipsilateral “Probst” bundles that run parallel to the midline (Islam et al., 2009; Fenlon et al., 2015). Since this aberrant neuronal circuitry is retained throughout adulthood, it may contribute to ASD-like behaviors in mice.

Furthermore, both *draxin* KO and BTBR/J mice have abnormalities in the topographic organization of connections between the thalamus and the cortex (Fenlon et al., 2015; Shinmyo et al., 2015). This suggests that the alteration in cortical area patterning caused by the deletion of the *draxin* gene contributes to the previously observed sensory and behavioral deficits in BTBR/J mice (Moy et al., 2007; McFarlane et al., 2008). It is critical to generate conditional *draxin* KO mice with specific neural structural abnormalities and perform behavioral analyses to investigate these possibilities. Recently, it was reported that BTBR TF/ArtRbrc (BTBR/R) mice, a sister strain of BTBR/J, show core symptoms of ASD despite having an intact *draxin* gene and preserved forebrain commissures (Lin et al., 2023). BTBR/R mice will be useful for understanding the draxin-independent mechanisms that cause ASD-like behaviors.
